# Depression and emotion regulation strategy use moderate age-related attentional positivity bias

**DOI:** 10.3389/fpsyg.2024.1427480

**Published:** 2024-12-16

**Authors:** Leonard Faul, Lucas Bellaiche, David J. Madden, Moria J. Smoski, Kevin S. LaBar

**Affiliations:** ^1^Center for Cognitive Neuroscience, Duke University, Durham, NC, United States; ^2^Department of Psychiatry and Behavioral Sciences, Duke University Medical Center, Durham, NC, United States

**Keywords:** eye-tracking, attentional bias, aging, emotion regulation, depression

## Abstract

Effective emotion regulation is critical for maintaining emotional health in the face of adverse events that accumulate over the lifespan. These abilities are thought to be generally maintained in older adults, accompanied by the emergence of attentional biases to positive information. Such age-related positivity biases, however, are not always reported and may be moderated by individual differences in affective vulnerabilities and competencies, such as those related to dispositional negative affect and emotion regulation styles. To examine these relationships, we analyzed eye-tracking data from 72 participants (35–74 years; 50 female), 44 without and 28 with a diagnosis of Major Depressive Disorder during a free-viewing task comprising neutral-neutral, negative-neutral, and positive-neutral image pairs. Emotional bias scores were calculated based on the ratio of time spent dwelling on the emotional image vs. the neutral image in each emotional-neutral pair. Results indicate that healthy participants exhibited a stronger positivity bias than a negativity bias, whereas individuals with higher depressive symptom scores showed no difference. Next, we examined how age and emotion regulation strategy use (reappraisal vs. suppression, measured with the Emotion Regulation Questionnaire) impacted these effects. Individuals with Major Depressive Disorder did not exhibit a significant relationship between age and positivity bias. However, for healthy participants who self-reported a preference for using reappraisal in daily life, increased age was associated with an increased positivity bias. These findings indicate that the emergence of the positivity effect in older adults is related to reappraisal regulatory preferences in the absence of depressive symptoms.

## Introduction

The degree to which an individual directs attention toward affective information interplays with their emotional health in crucial ways (Livingstone and Isaacowitz, 2017). For instance, attentional biases toward positive information are associated with higher rates of wellbeing (Blanco and Vazquez, 2021), whereas dwelling longer on negative information is associated with higher rumination and lower wellbeing in clinical depression (Holas et al., 2018). Much work has shown that one's affective state shapes attentional biases and general cognitive styles (Storbeck and Clore, 2008), which can in turn perpetuate negative affect or support positive affect depending on the focus of attention. Thus, where we engage visual attention has lasting effects on emotional fulfillment and mental health. Accordingly, a growing body of work has sought to detail the neurobehavioral mechanisms that govern these effects (Keller et al., 2019).

Research in this area typically assesses gaze preferences by tracking participants' eye movements and fixations (Armstrong and Olatunji, 2012). This work has revealed that attentional biases are shaped by a complex interaction of contextual cues, personal traits, and goals. The effect of age is particularly compelling, as increased age is associated with biased attention toward positive information—known as the attentional positivity effect—that facilitates concomitant improvements in emotional wellbeing (Mather and Carstensen, 2005). However, as prevalent as this increased positivity effect is in the emotion and aging literature, recent reviews have begun to highlight methodological and theoretical issues in this area (e.g., Isaacowitz, 2022). One concern is that other moderators could play crucial yet understudied roles in shaping age-related affective biases. For instance, attentional deployment toward emotional stimuli may also be biased by differences in emotion processing stemming from depressive symptoms or by dispositional preferences in emotion regulation (ER); that is, the typical strategies that someone uses to regulate their emotions. Yet, while these effects have been studied independently, pinpointing how all these factors combine to shape attentional deployment remains an open area of investigation.

In the present study, we investigated attentional biases to emotional images in a free-viewing eye-tracking paradigm, while considering the influence of dispositional ER strategies and age, our two focal variables of interest. With an exploratory assessment we also examined the moderating role of depression. By considering all of the above in an unconstrained affective bias task, we are afforded insight into how such traits interact with one another to guide emotional engagement in real world encounters. Accordingly, in the proceeding sections, we first review existing evidence for dispositional ER tendencies, age, and depression as moderators of attentional biases. We then integrate these literatures to guide our hypotheses for how such traits may interact with one another to shape attention.

### Emotion regulation and dispositional strategy use

ER–the attempt to influence one's emotional state–is an essential feature of mental health, especially in response to stressors and adverse life events. ER relies on meaningful judgments of affective stimuli that subsequently guide cognition and behavior toward desired emotional goals. For instance, the ability to appropriately regulate emotions facilitates a wide range of mental health benefits (e.g., Thomas and Zolkoski, 2020; see Menefee et al., 2022), while difficulties in implementing ER strategies predict higher risk of psychopathologies such as anxiety, depression, and substance abuse disorders (Aldao et al., 2010; Nolen-Hoeksema et al., 2008). These impediments in ER likely predispose individuals to process emotional information differently, which subsequently feeds into attentional biases that further perpetuate symptoms of psychiatric disorders (Holas et al., 2018).

Importantly, ER strategies vary widely in terms of cognitive load, (mal)adaptive tendencies, and timing of implementation (Kobylińska and Kusev, 2019). To account for these different modes of ER, Gross (1998) posited a seminal process model of ER that distinguishes between five successive classes: selection of situation, modification of situation, attentional deployment, cognitive change, and modulation of response. Much of the literature specifically highlights cognitive change, given its general efficacy in changing the trajectory of negative emotional response (e.g., Ochsner and Gross, 2008, 2005). Otherwise known as reappraisal, this strategy involves reinterpreting emotional situations to alter their emotional meaning (Lazarus and Alfert, 1964), such as reframing a job rejection as a valuable learning experience and an opportunity to pursue alternative career paths. However, reappraisal involves complex interactions among memory, language, goal-setting, and mental imagery processes. While these processes can become more automated with practice, emotion regulation via reappraisal has been shown to be more cognitively taxing than other strategies, like emotional acceptance (Keng et al., 2013).

In contrast to reappraisal, expressive suppression (a form of response modulation) is typically an inhibiting behavioral response that occurs following an emotional event, such as withholding negative expression while watching a car crash (Gross, 1998). Suppression does not alter the emotion generative process itself like other forms of ER, but rather the behavioral outcomes of the emotion. In contrast to reappraisal, higher rates of suppression are often associated with more negative reports of emotions (John and Gross, 2004), high-risk attachment styles (Gross, 2008), and worsened wellbeing and psychopathologies (Aldao and Nolen-Hoeksema, 2012; Gross and John, 2003; Kobylińska and Kusev, 2019). Collectively, these findings suggest that reappraisal may be a generally healthier and more effective method of ER than suppression, though variation exists across individuals, cultures, and contexts (Kraus and Kitayama, 2019; Soto et al., 2011).

To measure dispositional usage of reappraisal and suppression, Gross and John (2003) developed the self-report Emotional Regulation Questionnaire (ERQ) which provides composite scores for each of these two ER strategies. This self-reported index of dispositional ER represents the degree to which individuals use suppression and reappraisal in daily life, with higher reappraisal and lower suppression use often predicting better mental health (Moore et al., 2008). Individuals who report higher dispositional use of suppression and lower use of reappraisal have also been shown to exhibit differences on attention tasks by dwelling longer on threatening images (when paired with neutral images), suggesting an interplay between chronic suppression usage and attentional engagement (Bardeen and Daniel, 2017). While this relationship is correlational, it is possible that dispositional ER strategies have an influence on positive and negative attentional biases beyond just threat. The lack of research in this area is surprising, given that ER use has been shown to map onto emotional wellbeing, and yet its moderating role on attentional biases to emotional content remains less clear.

It is also important to note the finding from Bardeen and Daniel (2017) that attentional biases are especially pronounced for individuals high in suppression use *and* low in reappraisal use. Although the original development of the ERQ indicated that reappraisal and suppression were uncorrelated, this is not a consistent observation. Several studies have observed a negative correlation between these two regulation strategies, with the suggestion that practice with reappraisal can, over time, help to reduce reliance on suppression (e.g., Beaumont et al., 2023; Gullone and Taffe, 2012). It is also possible that the presence of psychopathological symptoms shifts the extent to which these strategies are correlated. A recent meta-analysis of emotion regulation in current and remitted depression, for instance, found that current depression is characterized by more maladaptive and less adaptive strategies compared to healthy controls, whereas remitted depression is only associated with more maladaptive strategies (Visted et al., 2018). The authors suggested that vulnerability to depressive relapse may be marked by the hindrance of adaptive strategies, such as reappraisal, by co-occurring maladaptive strategies, such as suppression. Similarly, cluster analysis of emotional subtypes in depression shows that intermediate levels of depressive symptomatology are associated with high levels of both reappraisal and suppression use (Chan et al., 2023). Alternatively, related work has indicated that a preference for reappraisal (high use of reappraisal and low use of suppression) is associated with the lowest levels of psychopathological symptoms, when compared to individuals who are high in both, low in both, or only moderately use reappraisal (Eftekhari et al., 2009). Collectively, then, these findings suggest that examining reappraisal and suppression use in tandem (e.g., a *preference* for one over the other) may provide a more holistic measure for how dispositional ER maps onto both psychological wellbeing and affective biases.

### The positivity effect in older adults

Age is another crucial factor that moderates attentional biases toward emotional material. Referred to as the positivity effect (Mather and Carstensen, 2005), older adults tend to prioritize positive information more so than younger adults, at least under some circumstances (Carstensen et al., 2003; Isaacowitz, 2022, 2012; Isaacowitz and Choi, 2011; Noh et al., 2011). Supporting this biasing effect, positive content in laboratory studies tends to be attended to and remembered more than negative content among older, relative to younger, adults (Isaacowitz et al., 2006; Mather and Knight, 2005; Rubin and Schulkind, 1997). These findings are particularly striking given that general cognitive declines in older adults could impede effortful ER implementation that one would predict to yield reduced negative and enhanced positive experiences (see Mather, 2012, for further discussion).

Given this paradox, then, there is debate as to how and why this biasing toward positive information specifically emerges (Isaacowitz, 2022). One major hypothesis used to explain differential positive consumption across age is that of attentional selectivity. Rather than being an outcome of more successful ER, positivity effects may be a consequence of behavioral motivations to specifically select more positive and personally satisfying situations for the focus of attention (Sims and Carstensen, 2014). Additionally, age-related cognitive changes may not necessarily alter the *success* of regulation as previously implied, but rather the *relationship* between attention to emotional information and subsequent implementation choice of certain strategies. For instance, younger adults are more likely than older adults to benefit from attending to negative stimuli when constructing reappraisal narratives (Bebko et al., 2011; Isaacowitz and Noh, 2011). However, this effect is diminished across age, as older adults are less successful in using reappraisal when gazing at negative images compared to younger adults (Opitz et al., 2012), and overall tend to look less at negative images when reappraising (Martins et al., 2018; Noh et al., 2011; van Reekum et al., 2007). Investigations into reappraisal use across age find that older adults engage with and benefit more from *positive reappraisal*, in which positive aspects of an experience are recognized. This process is often referred to as “benefit finding” and involves seeking for silver linings of negative situations, like focusing on the impressive artistic quality of negative film clips or emphasizing community building after tragic events. In contrast, younger adults are more adept at implementing *detached reappraisal*, which reduces the intensity of negative emotions by considering non-emotional aspects (i.e., “turn down the emotional volume”), like identifying familiar actors in negative film clips or considering practical next steps (Shiota and Levenson, 2009, 2012). Thus, while it seems that younger and older adults can implement ER strategies with similar success (e.g., Livingstone and Isaacowitz, 2018), there may be a shift in the relationship between attentional focus (i.e., toward more positive information) and ER implementation with age.

The question remains, then, when and why older adults would select to attend to positive information in the first place. Theoretical models like the Socioemotional Selectivity Theory (SST; Carstensen et al., 1999) implicate shifted mortality and temporal perspectives that guide positive biasing in aging, while the Strength and Vulnerability Integration Model (SAVI; Charles, 2010) emphasizes that accrued self-knowledge and lived experiences over time facilitate greater emotional wellbeing with age and guide cognitive resources toward positive information (Charles, 2010). When unconstrained in their attentional deployment, older adults look away from negative situations (e.g., avoiding interpersonal conflict; Birditt and Fingerman, 2005) and toward positive information (Knight et al., 2007), which then supports successful ER and dampened emotional reactivity (Charles et al., 2009; Hart and Charles, 2013). However, when faced with an unavoidable negative situation or stressor overload, these age-related positivity effects are diminished, emphasizing that the prioritization of positive information is rooted in attentional selectivity mechanisms that have been learned over time to mitigate daily negative affect (Charles and Luong, 2013). Crucially, however, age-related positivity effects are not ubiquitous. Some studies have found little to no evidence of positivity biases in older adults (see Reed and Carstensen, 2012; e.g., Gallo et al., 2009; Grühn et al., 2005). Mounting evidence points toward a multitude of cognitive and behavioral factors that may moderate the emergence of a positivity bias (Isaacowitz, 2022; Reed et al., 2014). For instance, Li et al. (2011) discovered smaller pupillary changes (indicating reduced emotional reactivity) in response to negative stimuli only in older adults who reported a higher use of reappraisal in daily life, providing preliminary evidence for the moderation of age-related affective biases depending on dispositional ER use.

In summary, increased age is associated with a prioritization of positive information and accrued life experiences/knowledge, underscored by a shift in the relationship between attentional deployment and strategy implementation for successful ER. Importantly, though, age-related positivity effects may be moderated by the constraints of the task at hand and individual difference factors including dispositional ER usage. However, to date, very few studies have investigated how dispositional ER tendencies and age are associated with biased responding toward emotional information.

### The role of depressive symptoms in further shaping attentional biases

As predicted by the SAVI model, older adults who have emotional vulnerabilities may not exhibit or benefit from attentional biasing toward positive information. Mood disorders like Major Depressive Disorder (MDD) have notable alterations in emotion processing, including greater attention toward negative information (e.g., Mennen et al., 2019; Peckham et al., 2010; see Armstrong and Olatunji, 2012 for a review). Similarly, others have found an association between depression and reduced viewing of positive images (Sears et al., 2011), or some combination of enhanced negative and reduced positive attention (Duque and Vázquez, 2015). By one common interpretation, these results are hypothesized to be related to a mood-congruent processing bias and self-verification of depressed mood (Arens and Stangier, 2020; Beck, 1976). In other words, depressed individuals hold an attentional bias toward negative information. Alternatively, according to the depressive realism hypothesis, depressed individuals may hold a more accurate truth-seeking outlook that diminishes the presence of positive biases rather than enhancing negativity biases (Haaga and Beck, 1995). Indeed, some work suggests this to be the case in certain contexts, as summarized in a meta-analysis by Moore and Fresco (2012). However, other studies have found that the depressive realism hypothesis may only hold below a certain level of depressive symptoms, and that above this threshold, negativity biases emerge in line with the mood-congruent argument (Korn et al., 2014; Szu-Ting Fu et al., 2012). Yet, the degree to which these competing hypotheses have been evaluated in the context of naturalistic attention toward emotional information remains relatively scarce, particularly in contrast to other, more popular paradigms leveraged in the depressive realism literature such as self-reflection and judgment tasks.

When considering depression across the lifespan, attentional biases in depression have been shown to exist irrespective of age (Lu et al., 2017), although a meta-analytical review of eye-tracking investigations with depressed patients observed that older participants do demonstrate a smaller difference in attention to positive content when compared between depressed and non-depressed groups (Suslow et al., 2020). However, these studies generally examined younger-middle aged adults (median age of 37 years), and most assessed attentional biases toward faces rather than emotional scenes. As such, more work is needed assessing attentional biases that shift with depressive symptoms across a wider range of ages and with paradigms utilizing more affectively engaging stimuli.

Lastly, depression is also marked by maladaptive ER practices, including increased rumination on negative experiences and higher usage of suppression and avoidance strategies in attempts to reduce negative affect (Aldao et al., 2010; Moore et al., 2008; Nezlek and Kuppens, 2008). While this link between depressive symptoms and maladaptive ER seems to generally hold across the lifespan (Nolen-Hoeksema and Aldao, 2011), increased age is associated with less variability in how specific strategies are used (de la Fuente et al., 2024). Interestingly, depression is not necessarily associated with the impaired ability to *implement* strategies such as reappraisal when patients are instructed to do so, but rather difficulties *selecting* the appropriate strategy for a given situation (Liu and Thompson, 2017). Although depressed individuals exhibit underutilization of cognitive reappraisal, this is not necessarily always accompanied by more self-reported suppression, as evidenced by mixed support for increased suppression in clinical depression (Dryman and Heimberg, 2018). Nevertheless, as noted previously, higher depressive symptoms seem to be particularly associated with less of a *preference* for reappraisal use (Chan et al., 2023). More work is needed, though, to clarify how reappraisal and suppression usage are associated with one another among individuals experiencing higher depressive symptoms, as well as whether regulatory preferences in depression are associated with attentional biases in the same way as in non-depressed individuals.

### Approach and hypotheses for the present study

Our review of these literatures identified several key moderators of attention that are often studied independently, yet have potential overlap. First, the way by which individuals prefer to regulate their emotions influences how emotional information is attended to and processed. Second, although studies have shown an age-related positivity effect on attention, the underlying mechanisms remain unclear and this finding is not consistently observed. Importantly, though, growing evidence suggests that changes in ER styles with age may facilitate the positivity effect. Third, depression is associated with differences in both attention and ER usage, which seem to hold across the lifespan. Thus, the presence of depressive symptoms may uniquely alter the relations among these variables. How, then, does age interact with ER preferences to shape attentional biases toward emotional information, and does the presence of depressive symptoms further moderate this interaction?

**Figure 1:**
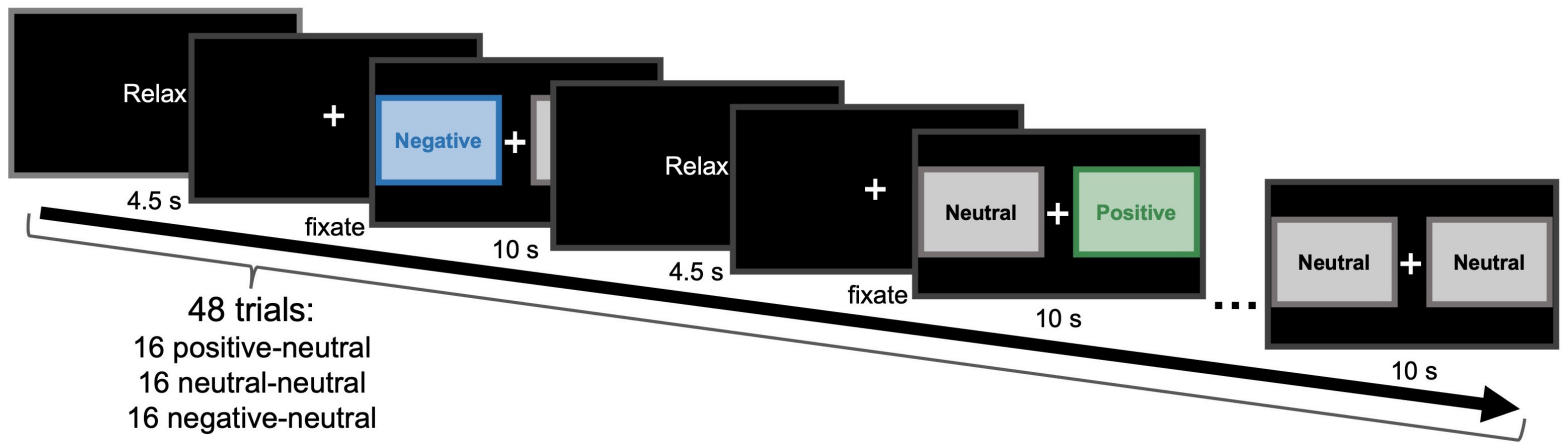
Task paradigm. Participants were sequentially presented with 48 trials. Each trial consisted of a relax period (4.5 seconds), a fixation period that lasted until the eye tracker successfully captured both eyes looking at the fixation cross, and a pair of images (10 seconds) taken from a 96-item subset of the IAPS repository. Participants could freely gaze between the two images. The 48 pairs consisted of 16 positive-neutral, 16 neutral-neutral, and 16 negative-neutral pairings, presented in random sequence. On each trial, the location of the images on the screen (left or right) was randomly assigned.

Given that no study, to our knowledge, has directly assessed this question, we aimed to fill this gap in the literature. We implemented an eye-tracking paradigm involving naturalistic viewing of positive, negative, and neutral images across an adult lifespan sample (Figure 1). Our sample also included individuals with and without Major Depressive Disorder (MDD) varying in depression severity to allow for an assessment into the role of depressive symptoms in further shaping affective attentional biases. This assessment, however, should be noted as exploratory given our smaller sample size for MDD participants.

We hypothesized that all participants would, in general, exhibit biased attention toward emotional over neutral images. We further hypothesized that this emotional bias would be moderated by age, such that increased age would be associated with more time proportionally viewing positive images and less time proportionally viewing negative images. When examining the role of depression, we expected an association between increased depressive symptoms and a more negative attentional bias, either due to increased viewing of negative images, decreased viewing of positive images, or a combination of both effects. Crucially, our primary analysis investigated whether a dispositional *preference* for reappraisal or suppression use moderates attentional biases toward emotional information, informed by prior work demonstrating specific mental health trajectories and attentional biases for individuals who predominantly use one ER strategy over the other. Given the benefits of reappraisal over suppression in regulating emotions, we hypothesized that a dispositional preference for using reappraisal in daily life would be associated with stronger positivity biases, especially for older adults who tend to regulate by disengaging from negative information and engaging with positive information. Collectively, our approach provides a novel assessment of how age and regulatory practices interact when considered in the context of attentional biases to emotional material, and how these may be further altered by the presence of depressive symptoms.

## Materials and methods

### Participants

The present investigation is part of a larger pre-registered study examining the neurobehavioral mechanisms of emotion regulation in aging and depression (registered as NCT03207503 at clinicaltrials.gov). Participants completed a battery of questionnaires, including the Beck Depression Inventory (BDI; Beck et al., 1961) and Emotion Regulation Questionnaire (ERQ; Gross and John, 2003), cognitive assessments of executive function and task-switching ability, eye-tracking while viewing emotional pictures (affective bias task), and functional neuroimaging during autobiographical memory recall. Our primary aim in the analyses reported here was to examine attentional biases toward emotional information as a function of age and emotion regulation across participants varying in depressive symptom severity, and thus we focus specifically on the data from the eye-tracking affective bias task.

English-speaking participants were recruited from the community by flyers, online advertisements, and recruitment messages through medical center communications from 2017 to 2022 in the Durham, North Carolina area. Inclusion criteria were an age range of 35–75 years, no MRI contraindications, no known neurological conditions or history of stroke, stable (or no) use of antidepressants or other psychotropics in the past 4 weeks, no known uncorrected sensory deficits, an estimated verbal IQ > 85 (assessed with the National Adult Reading Test, Nelson, 1982), and no indication of dementia or Mild Cognitive Impairment, as indicated by neuropsychological screening. Specifically, at an initial screening session, individuals who may have prodromal dementia were screened out based on performance on four neuropsychological measures: the Montreal Cognitive Assessment (MoCA; Nasreddine et al., 2005), Hopkins Verbal Learning Test-Revised (HVLT-R; Brandt and Benedict, 2001), Trail Making Test (Reitan, 1992), and 1-min Animal Naming (Lezak et al., 2012). To pass screening, the MoCA threshold for participation was set at 24 or above, while the HVLT-R, Trail Making, and Animal Naming thresholds were set at −1.5 SD below the mean based on age-corrected normative values (Brandt and Benedict, 2001; Heaton et al., 2004). Finally, participants with MDD were identified based on a SCID-5 (First et al., 2015) diagnosis of current MDD (single, recurrent, or persistent depressive disorder), BDI-II score of 14 or higher at initial screening, no current substance use disorder, and no history of mania, psychosis, or eating disorder. Control participants had no past or present Axis I psychopathology and a BDI-II score of 8 or lower at initial screening. Note, however, that for our analyses incorporating BDI scores, we used scores obtained from a second BDI assessment obtained closer to the date of the eye-tracking affective bias task, which resulted in one participant with MDD scoring below 14 (control BDI range = 0–8; MDD BDI range = 10–47).^1^

Eighty-two participants passed this initial screening phase and also performed the eye-tracking affective bias task. However, we excluded 10 of these participants (age range 37–70 years old, median age = 53, 5F, 2 MDD) from all analyses for the following reasons: four participants were excluded due to technical difficulties with the eye-tracker keeping track of both eyes throughout the entire task, one participant's data was not saved due to experimenter error, and an additional five participants were excluded due to significant data loss (>33% of trials) after preprocessing the eye-tracking data (see below). The final sample consisted of 72 participants, with 44 healthy controls (35–74 years old, median age = 56; 27F, 17M) and 28 participants with MDD (35–68 years old, median age = 49; 23F, 5M). See Supplementary Figure S2 for distributions of participant ages.

The control group (2 Hispanic or Latinx, 42 not Hispanic or Latinx) included 1 Asian participant, 36 Caucasian participants, 4 Black or African American participants, 1 Native Hawaiian or Pacific Islander participant, and 2 participants who identified as a race not included in the list. The MDD group (1 Hispanic or Latinx, 27 not Hispanic or Latinx) included 1 Asian participant, 21 Caucasian participants, 5 Black or African American participants, and 1 participant who identified as a race not included in the list. Fifteen of the MDD participants reported stable use of antidepressants or other psychotropics. Across both groups, a majority of participants were college graduates (*n* = 62), many of whom also completed additional graduate school training (*n* = 45). All participants completed the ERQ in a separate online session (Gross and John, 2003). All participants provided written informed consent in accordance with the Duke University Health System Institutional Review Board and were monetarily compensated for their time ($65 for interview/assessments/questionnaires and $35 for task completion).

While a power analysis was conducted for the primary statistical model tested by the broader pre-registered study, we did not perform a separate *a priori* power analysis for this specific assessment on attentional bias, although our sample size is comparable to or exceeds similar work in this area (e.g., Duque and Vázquez, 2015; Holas et al., 2018; Isaacowitz et al., 2006; Li et al., 2011; Noh et al., 2011; Sears et al., 2011; van Reekum et al., 2007; see Table 1 of Armstrong and Olatunji, 2012). Using G^*^Power (Faul et al., 2007), we also determined that with our total sample size (*n* = 72), the power to detect a repeated measures, within-between interaction (two groups by two conditions/measurements) with an effect size of η^2^ = 0.12 (based on previous findings from Duque and Vázquez, 2015, and Sears et al., 2011 on attentional biases between depressed and non-depressed groups), was 0.87 at an alpha of *p* = 0.05 (using the Cohen effect size setting). Our primary analyses, however, examined the three-way interaction of age, emotion condition, and ER preference separately in the control and MDD groups. Approximating such a design with G^*^Power indicates that these analyses are sufficiently powered to detect a large effect size, but may be underpowered to detect smaller effect sizes (see Supplementary material for more detail).

Importantly, though, note that our analyses employed a more sensitive linear mixed-effects regression than G^*^Power estimations can appreciate. We used both categorical (e.g., emotion condition) and continuous (e.g., age, dispositional ER, and BDI score) fixed effects while subjects were specified as random effects. In this way, we utilized all available datapoints across all trials in the analyses (total *n* = 3,299 across all conditions). The aforementioned power estimations are from the most representative models allowed by G^*^Power for the analyses that were performed here. Nevertheless, we acknowledge that our sample size remains underpowered to detect small and medium effect sizes for higher-level interactions. Given that we did not perform an a priori power analysis for this specific objective, and due to the limitations of *post-hoc* power analyses, we report effects with confidence intervals to aid in the interpretation of our findings (Dziak et al., 2020).

### Affective bias task

The affective bias task consisted of participants freely viewing image pairs for 10 seconds each over a total of 48 trials, with each trial consisting of either a neutral–neutral, negative–neutral, or positive–neutral pair (Figure 1). This visual paired-comparison task expanded upon LaBar et al. (2001) and was chosen because age-associated attentional positivity biases tend to be more prominent with longer viewing times (Isaacowitz et al., 2009) and because a naturalistic, free-viewing task reduces experimenter demands on attentional allocation by allowing participants to decide where to look on the screen. Accordingly, 16 positive, 16 negative, and 64 neutral images were selected from the International Affective Picture System (IAPS; Lang et al., 2008), such that normative ratings dissociated the image sets on valence (*F*_2, 93_ = 469.887, *p* < 0.001) and arousal (*F*_2, 93_ = 9.735, *p* < 0.001), while minimizing any differences in red-green-blue color, luminosity, contrast, and complexity (all *p*s >0.05). Specifically, normative valence ratings were such that positive (mean = 7.58), neutral (mean = 4.97), and negative (mean = 2.61) images were all significantly different from each other in valence (all *p*s < 0.001). Normative arousal ratings indicated that positive (mean = 5.24) and neutral (mean = 4.31) images were significantly different from one another in arousal (b = 0.926, *t*_93_ = 4.112, Bonferroni-corrected *p* < 0.001, 95% CI = [0.377, 1.475]), while the difference between negative (mean = 4.85) and neutral images was marginally significant (b = 0.539, *t*_93_ = 2.391, Bonferroni-corrected *p* = 0.056, 95% CI = [−0.011, 1.088]). Positive and negative images did not differ in arousal (b = 0.388, *t*_93_ = 1.360, Bonferroni-corrected *p* = 0.531, 95% CI = [−0.307, 1.082]). Prior to the start of the task, two lists of images were randomly generated for each participant—one containing 48 randomly-ordered neutral images, and the other containing the remaining 16 neutral images along with the 16 positive and 16 negative images, also in a random order. These lists were combined to create pairs of images for each trial. For each pair, the two images were randomly assigned to be shown on either the left or right side of the screen.

During the task, images were presented on a computer monitor with a resolution of 1,280 × 1,024 pixels for a total of 10 seconds. Participants were seated approximately 60 cm in front of the computer screen, and head movements were minimized by use of a chin rest. Each image occupied a space of 576 × 346 pixels on the screen, separated by a fixation cross. Participants were instructed that they may look at the images in any way they wish during this time, but to not look away from the screen. Trials were separated by a screen showing the word “relax” for 4.5 seconds, followed by a fixation cross in the center of the screen that participants needed to fixate on before the next trial would begin. Fixation position was tracked with a Tobii T60 eye-tracker (Tobii Technology) sampling at 60 Hz or a Tobii Spectrum (Tobii Technology) also sampling at 60 Hz.

### Eye-tracking preprocessing

During data collection, tracked data points were sorted into left or right areas of interest (AOI) if the position of the fixation was inside the left or right image, respectively. During preprocessing, samples obtained in these AOIs were grouped together if they were obtained successively. That is, given that we sampled at 60 Hz, samples were placed into separate bins if they were separated by 33 milliseconds or more (2 or more samples). For 5 subjects, a technical error reduced the precision of timing information, requiring us to use a slightly higher threshold for identifying samples within an AOI that were obtained successively (66 or 133 ms). Once data were binned, we next identified bins that consisted of at least 100 ms of successive data points by computing the difference in time between the final sample and the first sample. Only bins consisting of at least 100 ms of data were used to calculate dwell times within each AOI. We identified and removed trials with poor eye-tracking data if the first sample recorded in a trial was already inside an AOI, given that our task code required the initial eye position to be centered on a fixation cross in-between the images at the start of each trial (and thus this discrepancy indicated difficulty tracking the eyes at trial onset). We further excluded trials if the latency to saccade to the first AOI was >3 SD from the average latency across participants (mean = 0.31 seconds, SD = 0.25), and/or if the total number of samples obtained in the trial was <3 SD from the average number of samples collected across participants (mean = 504, SD = 79).

### Analysis

All analyses were performed in R. To examine the effects of age, depression, and dispositional ER use on attentional bias, we used linear mixed-effects regression models fitted by maximum likelihood with the *lme4* package, version 1.1-33 (Bates et al., 2015). Subjects were specified as random effects, and significance for fixed effects was assessed using Satterthwaite approximations to degrees of freedom (Satterthwaite, 1941). Lower-level interactions and simple effects/slopes were examined with the *emmeans* package, version 1.8.5 (Lenth, 2023). We report standardized beta coefficients for all regression analyses.

## Results

### Positivity biases in attentional allocation to emotional stimuli

**Figure 2:**
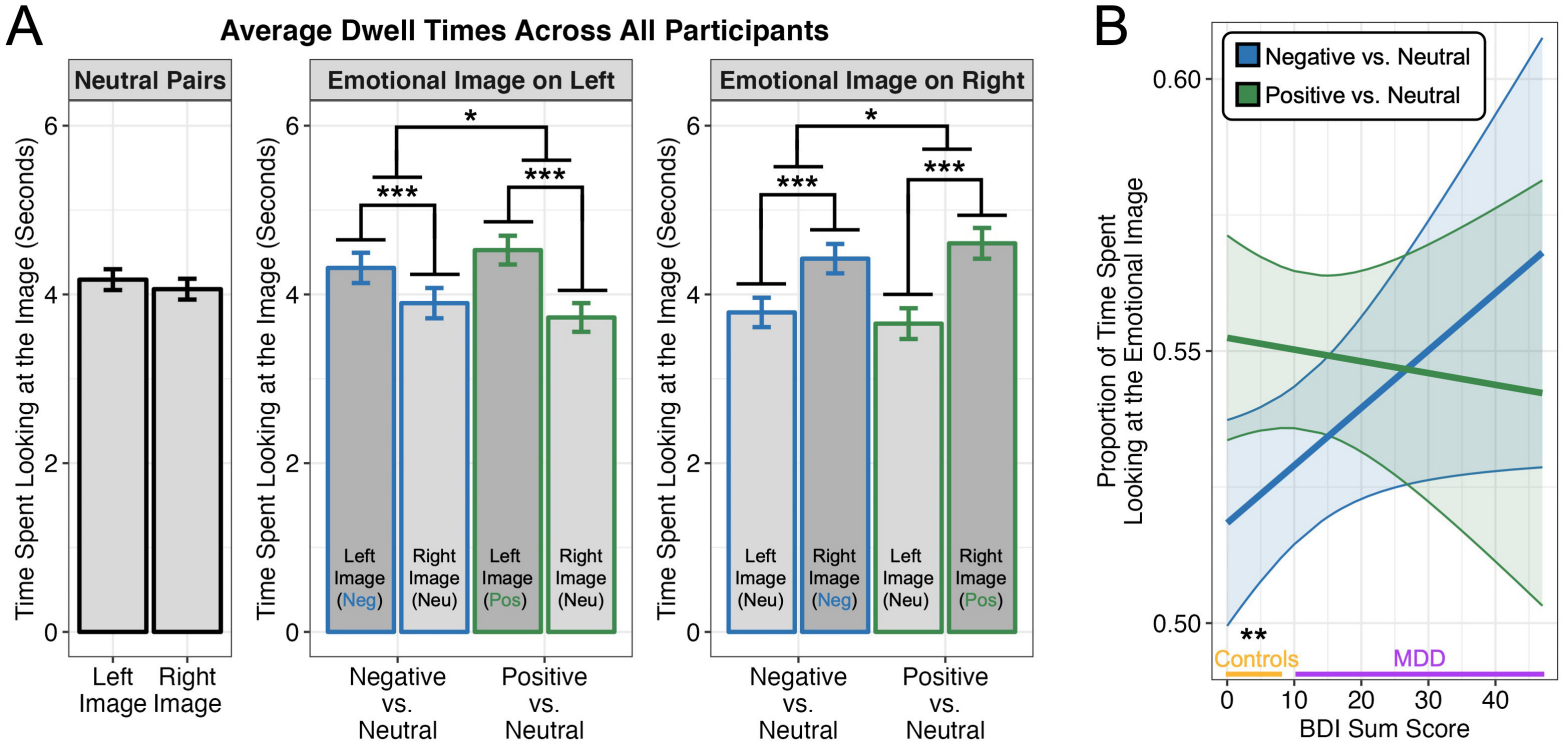
Dwell times on images as a function of emotion. **(A)** Overall, participants (*n* = 72) spent more time looking at the emotional than the neutral images in each pair, irrespective of spatial location (left or right). We also observed a significant interaction of image emotion and pair condition due to a stronger differential attentional bias toward positive images in the positive–neutral pairs relative to the attentional bias toward negative images in the negative–neutral pairs (middle and right panels). No difference in dwell time was observed as a function of spatial location for neutral-neutral image pairs (left panel). **(B)** Regression with Beck Depression Inventory (BDI) sum scores revealed that only participants with lower depressive symptoms exhibited a stronger attentional bias for positive–neutral pairs than negative–neutral pairs. On the x-axis, the range of BDI scores for individuals diagnosed with Major Depressive Disorder (MDD) are indicated in purple while those for healthy controls are indicated in orange. All plots depict estimated marginal means from the linear mixed effects model and 95% confidence intervals/bands. *p < 0.05, **p < 0.01, ***p < 0.001.

We first examined whether attention was generally biased toward the emotional images on the screen. For this analysis, we tested the three-way interaction of *image pair/condition* (positive–neutral or negative–neutral pair), *image location* (emotional image was on the left or on the right side of the screen), and *image emotion* (whether participants were looking at the emotional or neutral image within each pair) on dwell times (Figure 2A). Neutral–neutral pairs were not included in these analyses. We observed a main effect of image emotion (*F*_1, 4248.9_ = 158.482, *p* < 0.001), such that participants looked more at emotional images than neutral images (β = 0.37, *t*_4, 257_ = 12.579, *p* < 0.001, 95% CI = [0.312, 0.427]). This main effect was qualified by a significant interaction of image emotion and image pair (*F*_1, 4248.9_ = 9.727, *p* = 0.002), such that emotional biases were more prominent for positive–neutral than negative–neutral pair trials (β = 0.183, *t*_4, 257_ = 3.116, *p* = 0.002, 95% CI = [0.068, 0.298]). We observed no interactions with image location (all *p*s > 0.05), indicating that the main effect of image emotion and interaction with image pair did not depend on the spatial location of the emotional image on the screen (left or right). In a separate analysis, we did not observe an effect of image location on neutral–neutral pairs (*F*_1, 2063.3_ = 1.974, *p* = 0.160), indicating that participants exhibited similar dwell times for both left and right images in the absence of an emotional stimulus on the screen.

In sum, our initial analysis of the eye-tracking data indicated that participants were overall biased toward looking at emotional images more than neutral images when presented concurrently in mixed-valence pairs, but that this effect was stronger for positive images (attentional positivity bias).

### Depressive symptoms are associated with loss of a positivity bias

To explore a potential moderating role of depression, we tested the interaction of BDI sum scores with emotional image pair (positive–neutral or negative–neutral). For these analyses, we computed an emotional bias score by calculating the proportion of time spent viewing the emotional image vs. the total time spent viewing both images. Values above 0.5 therefore indicate an attentional bias toward the emotional image over the neutral one. We used BDI sum scores to test the effect of depression instead of a dichotomous group assignment, given that we had fewer MDD participants than controls, and regressing a continuous measure provides more power while also allowing us to examine effects that scale with the severity of depressive symptoms (control group BDI mean = 1.8, SD = 1.9, range = 0–8; MDD group BDI mean = 27.3, SD = 9.3, range = 10–47). This analysis revealed a significant interaction of depression levels and condition (*F*_1, 2093.3_ = 3.922, *p* = 0.048), which we unpacked by examining the difference in estimated marginal means between positive–neutral and negative–neutral conditions when marginal effects (estimated by our model) were averaged across the range of BDI values observed for control participants (0–8) and MDD participants (10–47). This approach revealed that only control participants exhibited an attentional positivity bias (β = 0.138, *t*_2, 096_ = 2.829, *p* = 0.005, 95% CI = [0.042, 0.234]), whereas depressed participants showed no difference between conditions (β = −0.011, *t*_2094_ = −0.163, *p* = 0.871, 95% CI = [−0.141, 0.120]). As shown in Figure 2B, the lack of a positivity bias was primarily driven by an increase in dwell times on negative images with increasing BDI scores (β = 0.070, *t*_183_ = 1.969, *p* = 0.051, 95% CI = [0.000, 0.140]).

### Healthy older adults exhibit a positivity effect only if they prefer to use cognitive reappraisal in daily life

We next tested whether older healthy adults would show the strongest bias for positive images (i.e., a positivity effect), and whether this bias would be amplified among those who prefer to reappraise in daily life. First, we examined the interactive effect of age, BDI, and image pair/condition (positive–neutral or negative–neutral) on dwell times, for which we did not observe a significant three-way interaction (F_1, 2093.44_ = 0.001, *p* = 0.981) or a significant two-way interaction of age and condition (F_1, 2094.07_ = 0.657, *p* = 0.418). For completeness, we also tested the moderating effect of age within the control and MDD groups separately. As before, the main effect of condition was only significant for controls (*F*_1, 1275.3_ = 6.656, *p* = 0.01), and not for MDD participants (*F*_1, 818.92_ = 0.028, *p* = 0.867). However, in neither of the groups did we observe a main effect of age (Controls: *F*_1, 45.48_ = 0.343, *p* = 0.561; MDD: *F*_1, 27.96_ = 0.022, *p* = 0.884), or an interaction of age and condition (Controls: *F*_1, 1275.45_ = 1.299, *p* = 0.255; MDD: *F*_1, 819.07_ = 0.021, *p* = 0.886). Thus, irrespective of depressive symptoms, older adults did not generally exhibit stronger positivity biases than younger adults.

**Figure 3:**
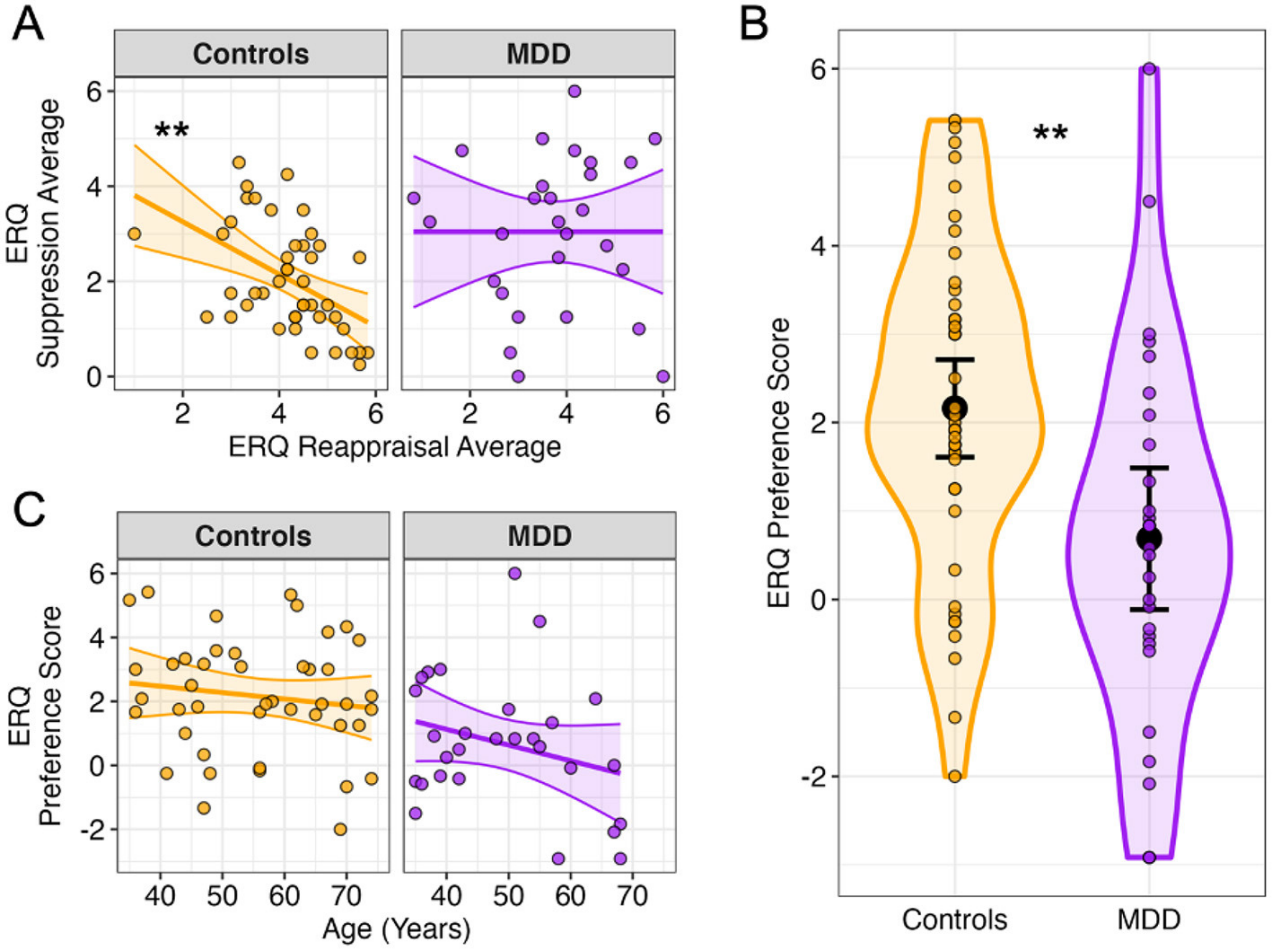
Emotion regulation strategy preferences in healthy and depressed individuals. **(A)** Reappraisal use is negatively associated with suppression use in healthy controls, but not MDD participants. **(B)** Healthy controls exhibit a general preference for reappraisal use, while MDD participants exhibit no preference for either strategy (ERQ preference score = reappraisal average – suppression average). **(C)** For both groups, age was not significantly associated with an ERQ preference for reappraisal or suppression. Plots depict averages for each participant and 95% confidence intervals/bands. **p < 0.01.

Importantly, though, we hypothesized that the degree to which participants prefer to use reappraisal vs. suppression in daily life would moderate dwell times. To test this proposal, we computed an emotion regulation preference score for each subject by subtracting the average score across suppression items from the average score across reappraisal items on the ERQ. Positive values therefore indicate a preference for using reappraisal in daily life, whereas negative values indicate a preference for using suppression, and a value of zero indicates equal endorsement of reappraisal and suppression use. When computing these scores, we observed that only control participants exhibited a significant negative correlation between reappraisal and suppression use (Figure 3A; *r* = −0.476, *p* = 0.001), but not MDD participants (*r* = 0, *p* = 1). A Fisher's z-test showed that these correlations were significantly different from one another (z = −2.04, *p* = 0.041). Accordingly, on average, control participants preferred using reappraisal more than MDD participants, who did not exhibit a bias for either strategy (*F*_1, 70_ = 10.144, *p* = 0.002; Figure 3B). Finally, we confirmed that the ERQ preference score was appropriate to test as a moderating variable on the relationship between age and dwell times, given that ERQ preferences and age were not significantly correlated with one another in control (*r* = −0.135, *p* = 0.382) or MDD participants (*r* = −0.274, *p* = 0.158), thus assuaging potential concerns of collinearity in the analysis (Figure 3C). See Supplementary Figure S1 for an overview of all correlations among the primary variables of interest for this analysis.

**Figure 4:**
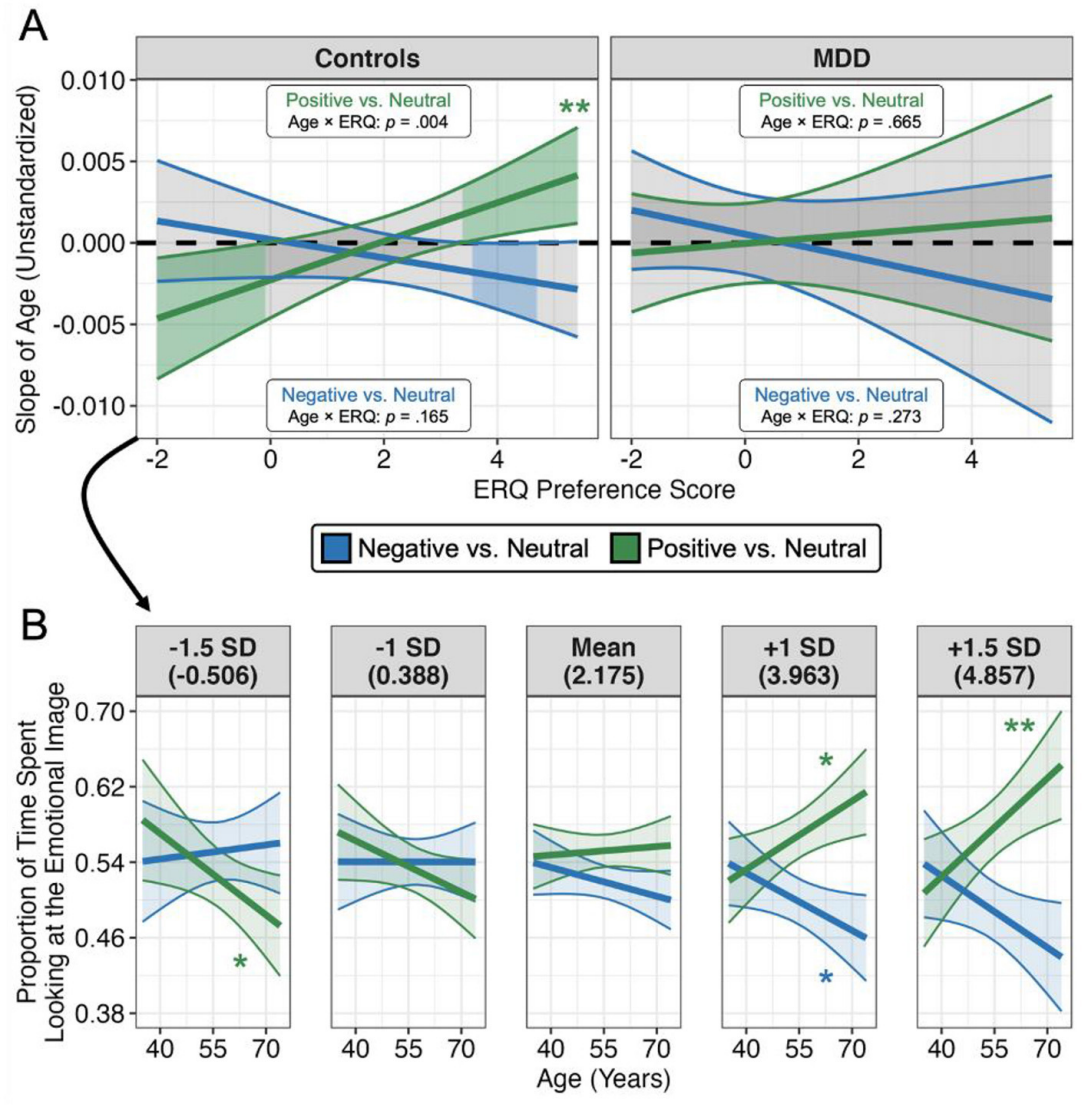
Emotion regulation style moderates age-related positivity biases in non-depressed individuals. **(A)** The relationship between age and dwell times (y-axis) shifts based on ERQ preference scores (x-axis). Regions where the regression line and confidence band do not cross zero indicate a significant (*p* < 0.05) slope of age. As ERQ preference score increases (more preference for using reappraisal than suppression), the relationship between age and dwell times becomes more positive for positive images, and more negative for negative images, but only in healthy controls. **(B)** The relationship between age and dwell times at specific levels of ERQ preference, only for healthy controls. Values in parentheses indicate the raw ERQ preference score at each level from −1.5 to +1.5 SD (standardized within controls). Both plots depict estimated marginal trends computed from the higher-level linear mixed effects model and 95% confidence intervals/bands. *p < 0.05, **p < 0.01.

Next, we tested the three-way interaction of age, condition, and ERQ preference on dwell times separately within the control and MDD groups, given the aforementioned depression-related differences in attentional biases and ERQ preference scores. We observed a significant three-way interaction for control participants (*F*_1, 1273.56_ = 11.395, *p* < 0.001), but not MDD participants (*F*_1, 819_ = 2.149, *p* = 0.143), which we unpacked by examining how the relationship between age and dwell times (i.e., the slope of age) changes with a preference for suppression or reappraisal in daily life. As shown in Figure 4A, increasing use of reappraisal over suppression in daily life was associated with a more positive relationship between age and dwell times for positive images, and a more negative relationship between age and dwell times for negative images. Accordingly, at a high preference for reappraisal use (+1 SD, 3.963), increased age was associated with dwelling more on positive images (Figure 4B; β = 0.139, *t*_147_ = 2.371, *p* = 0.019, 95% CI = [0.023, 0.255]) and dwelling less on negative images (β = −0.117, *t*_145_ = −2.000, *p* = 0.047, 95% CI = [−0.232, −0.001]). At a low preference for reappraisal use (−1 SD, 0.388), age was not associated with changes in dwell times for positive (β = −0.104, *t*_149_ = −1.715, *p* = 0.088, 95% CI = [−0.225, 0.016]) or negative (β = 0.000, *t*_149_ = −0.006, *p* = 0.995, 95% CI = [−0.121, 0.120]) images. In other words, a positivity bias only emerged for healthy older adults who prefer to use reappraisal in daily life (see Supplementary Figure S3 for an alternate view of this interaction with ERQ preference scores on the x-axis, shown at different levels of age). Similar, yet weaker, effects were observed when reappraisal and suppression use were assessed independently, indicating that the observed effects were indeed driven by a preference for reappraisal over suppression (see Supplemental material for more detail).

We note, however, that the four-way interaction of age, condition, ERQ preference, and BDI scores was found to be non-significant when assessed across all participants (F_1, 2091.82_ = 0.480, *p* = 0.489), indicating insufficient evidence for a statistically significant difference in the three-way interaction between groups (similar effects were found when using dichotomous group assignment instead of continuous BDI scores in the analyzed model). We caution, however, that our sample size was not appropriately powered to detect such a complex four-way interaction. We refrained from merely reporting the three-way interaction of age, condition, and ERQ preference across all participants (while controlling for the effects of BDI; F_1, 2092.20_ = 13.718, *p* < 0.001), as this approach may obscure potentially meaningful differences related to depression, and our findings have converged in demonstrating the impact of depression across multiple levels of analyses (e.g., depressed individuals exhibit a lack of a positivity bias in dwell times and lack an association between reappraisal and suppression usage). Moreover, even though the four-way interaction with BDI was not significant, the three-way interaction of BDI, condition, and ERQ preference (while controlling for age) was significant (see Supplementary Figure S4; F_1, 2091.44_ = 8.070, *p* = 0.005), with follow-up tests indicating that a higher preference for reappraisal was generally associated with a stronger positivity bias only in control, but not MDD, participants (irrespective of age; see Supplementary material for more details). Thus, our analyses consistently demonstrate that a preference for reappraisal or suppression use did not moderate attentional biases for individuals with depression, but did for non-depressed controls.

## Discussion

A rich combination of literatures suggests that where we divert our attention is characterized by a multitude of factors, including individual traits and emotional information. This study aimed to clarify the effect of some of these components and their potential interactions. Our findings overall supported our hypotheses and indicated that a positivity bias emerged and strengthened with increasing age (i.e., an age-related positivity effect), but only in healthy individuals who prefer using reappraisal-based regulation strategies in daily life. This finding extends the notion that, as individuals become older, attention toward positive (and away from negative) information is more associated with regulating affect (Bebko et al., 2011; Isaacowitz and Noh, 2011; van Reekum et al., 2007). Here, we show that dispositional reappraisal usage is also associated with positive attentional biases in healthy older adults. These results, then, provide nuance to the general age-related positivity effect by suggesting potential prerequisites in order to realize such an effect: older adults who utilize adaptive ER strategies like reappraisal more than maladaptive ones like suppression tend to show the strongest positivity effects, particularly for those without depression. The effects we observed here may also reconcile findings where age-related positivity effects were not supported as dispositional ER usage was not measured in these cases (e.g., Gallo et al., 2009; Grühn et al., 2005). Indeed, in the present study, an age-related positivity bias did not emerge without considering the interaction with ERQ preference scores.

Although our depression analysis was preliminary, our findings encourage future research at the intersection of age, depression, and regulation styles. Indeed, both age and clinical depression have been popular foci of emotion processing research, but few studies have considered how these factors may interact with one another and also interplay with ER practices to engender attentional affective biases. Neural circuitries of ER-related systems have been studied across the lifespan of both healthy and MDD participants (Aizenstein et al., 2011; Alexopoulos, 2005; de Asis et al., 2001; Tadayonnejad et al., 2014). Behaviorally, older patients with MDD have also had trouble regulating emotions across a series of paradigms (Orgeta, 2011; Smoski et al., 2014; Whitehead and Bergeman, 2014). However, minimal work has examined dispositional ER preferences across age and MDD on attentional biases, a key component of emotion processing and affective experience. While our findings do converge in part with past eye-tracking research indicating that older adults exhibit less pupil dilation (i.e., arousal) to negative images only if their dispositional use of reappraisal is high (Li et al., 2011), our study extends this work to attentional biases during naturalistic viewing and suggests an additional moderating impact of depression.

Specifically, the eye-tracking data and dispositional ER preferences from MDD patients were notable in their aberrant and non-significant associations on multiple counts, in line with other findings of irregular patterns of ER in MDD (Gross and Muñoz, 1995). Firstly, though non-depressed participants utilized reappraisal more than suppression and showed a negative association between usage of the two strategies, MDD participants did not display any such effects. ERQ preferences did not map onto the relationship between age and attentional bias in MDD participants as it did in healthy controls. That is, MDD was consistently associated with similar biases to both negative and positive information (lack of a positivity bias), irrespective of age or dispositional ER use. Further, that MDD participants exhibited these similar biases toward both positive and negative images may corroborate claims of the depressive realism literature (Haaga and Beck, 1995; Moore and Fresco, 2012). For instance, Korn et al. (2014) similarly showed that depression is associated with approximately equal rates of positive and negative information processing (as measured by belief updating tendencies from desirable and undesirable information, respectively), but that it is healthy controls who overly bias positive information. However, as in this study (Figure 2B), negativity biases emerged and strengthened linearly as depressive symptoms worsened, also mirroring results of the broader depression literature showcasing a switch from depressive realism toward negativity biases with increasing symptom severity (e.g., Szu-Ting Fu et al., 2012). Studies should continue to investigate at what degree of symptom severity this switch may occur, including in eye-tracking paradigms as in the current paper. Given the relatively low sample size of our MDD group, future research will also need to confirm whether the above effects replicate in larger samples that have more power to examine complex four-way interactions involving emotional valence, age, depression, and dispositional ER preference. Further, given that 15 of our 28 MDD participants reported some antidepressant or psychotropic usage, it would be interesting to assess how heterogeneity in medication may modulate findings in larger samples that have the power to explore this potential moderator.

In addition, the results stemming from the ERQ—that healthy adults exhibit a preference for dispositional use of reappraisal more than suppression in daily life, while there were more sporadic patterns in depression—brings into question the manner in which dispositional ER usage is measured. Many ER studies in the literature tend to examine reappraisal and suppression separately, without considering preferences for one strategy over another. Our findings illustrate that this preference score is reflective of the opposing relations that reappraisal and suppression often have with emotional outcomes, and motivate further study of preference scores as an indicator of individual differences in regulatory styles. Although the initial development of the ERQ proposed that the reappraisal and suppression factors were independent of one another (Gross and John, 2003), these relations may shift depending on psychopathology (as suggested here). Indeed, our findings align with prior work showing that reappraisal and suppression use are more likely to be co-endorsed among individuals with higher psychopathological symptoms (Chan et al., 2023; Eftekhari et al., 2009; Visted et al., 2018). Accordingly, we have shown here that only older adults who prefer to use reappraisal over suppression show an attentional positivity bias, in the absence of depression. Conversely, when older adults prefer suppression, attention toward positive images is actually reduced compared to younger adults. It is not readily clear why some older adults preferred to use reappraisal in daily life whereas others preferred suppression, but these preferences likely map onto individual differences in personality traits and life experiences that were outside the scope of our investigation (Eldesouky and English, 2019). An interesting avenue for future research will be to more clearly define how other individual differences, including physical health and social factors, shape regulatory preferences into later adulthood.

It is also important to note that this moderating effect of ERQ preference on attentional biases was specific to the older adults in our sample. That is, significant effects (diverging slopes) were localized to the right half of each panel in Figure 4B (see also Supplementary Figure S3 for an overview of effects displayed at different age levels). The specificity of this moderation dovetails with other findings that younger adults pay more attention to negative content when constructing reappraisal narratives (Isaacowitz and Noh, 2011; Bebko et al., 2011), and that older adults benefit more from positive reappraisal usage (Shiota and Levenson, 2009). Thus, even if younger adults habitually prefer to use reappraisal in daily life, this does not seem to map onto positive attentional biases in the same way as with older adults. It must be noted, however, that our sample used a cross-sectional design, which limits inferences about trajectories of change with advancing age. In addition, it is possible that we did not observe emotional attentional biases in younger adults given that the minimum age in our sample was 35. Negative biases may be strongest among younger cohorts that are more typically assessed in the literature (e.g., college-aged adults).

Moreover, it remains unclear the exact role of ER in this task design. Because we measured dispositional ER use with the ERQ, we do not know the extent to which self-reported, preferential use of ER strategies accurately depict how our participants would regulate their emotions to acute stressors. Recent work, however, has suggested that self-reported dispositional use of reappraisal does actually map onto successful implementation of reappraisal techniques (Wylie et al., 2023). Nevertheless, we were unable to determine if participants were directly implementing these strategies when engaging in the free-viewing paradigm. Subjects weren't *instructed* to use reappraisal or suppression, so any effects on attentional gaze allocation may be more implicit in nature, although we did not ask participants during debriefing whether they intentionally used specific strategies during the task. Because ER strategies were not sampled during the task itself and due to the correlational nature of the data, we must be cautious to imply that dispositional reappraisal alone led to attentional differences with advanced age. ER strategies can co-occur (Ford et al., 2019; Kobylińska and Kusev, 2019), and, as such, participants may have engaged in additional modes of ER during eye-tracking, including, most obviously, attentional deployment. It must also be noted that the lines between attentional deployment, suppression, and reappraisal may not be as distinct as originally proposed in the Gross (1998) process model. Bebko et al. (2014) claimed that even though attentional deployment can be engaged in isolation, attentional deployment also cognitively underlies both reappraisal and suppression. That is, reappraisal involves shifting attention from one interpretation of an emotional event to another reappraised one, and suppression deploys attention toward an inhibiting emotional response rather than toward the emotional stimulus itself. Thus, the specific success behind either reappraisal or suppression is not strictly due to attentional deployment (as both share this), but rather processes specific to each.

That said, there may be differences in executive functioning that also shape these effects. Reappraisal use is associated with executive functioning ability (including updating, shifting, and inhibitory processes), given that reappraisal involves a conscious attempt to override and reinterpret prevalent emotional signals (Toh et al., 2024). In contrast, suppression primarily involves inhibitory control and is generally a less cognitively taxing process (Goldin et al., 2008; Gyurak et al., 2012). One may reasonably speculate, then, that reappraisal recruits greater total executive functioning in comparison to suppression (Cohen et al., 2012). Thus, it is possible that the results reported in this manuscript are a manifestation of differing executive functioning abilities among older adults rather than emotion-related processes specific to ER. This interpretation is in line with prior work suggesting that the age-related positivity effect is more prominent among older adults with better cognitive abilities (Mather and Carstensen, 2005).

Regardless of the exact mechanisms if and by which participants were engaging in ER during the eye-tracking task itself, an association nonetheless emerges between the positive preferences elicited by attentional deployment in healthy older adults and their preference for reappraisal (as opposed to suppression) in their day-to-day life. Future research should clarify the interactions among these various regulatory strategies. Moreover, given these findings, it is a compelling possibility that interventions designed to decrease a dispositional preference for suppression use are particularly helpful for older adults, as this may facilitate greater focus on positive content and less focus on negative content.

While our findings imply a novel and unique pattern underlying affective attentional biases, we note some limitations of the current study. This includes the sample size (particularly for our depressed sample which was relatively small compared to the healthy controls) and the somewhat limited age range across the adult lifespan. The lower number of MDD participants was a consequence of the COVID-19 pandemic, our pre-screening requirements, and the general difficulty in recruiting older participants with MDD who passed inclusionary criteria for other facets of the larger study (e.g., MRI safety). Our depression-related analyses consistently displayed effects in line with the literature (i.e., the loss of a positivity bias and lower dispositional reappraisal use), and the attentional bias results were observed when depression was sampled as a continuous variable across all participants. Nevertheless, future research is needed to confirm whether these findings replicate in a larger depressed sample. In addition, while our sample was focused on testing the role of age on attentional biases, future work should examine other potentially meaningful individual differences such as education, socioeconomic status, and cultural influences.

Finally, the stimuli were taken from the IAPS repository, which has been normed and validated as a stimulus set across methodologies and is frequently used in eye-tracking designs (Isaacowitz and Choi, 2011; Lang et al., 2008), but the images presented to participants were not normed on social cues. It is possible that participants may have been influenced by potential pairings of social vs. non-social images, particularly across age and depression status, which are especially sensitive to social information (Luong et al., 2011; Segrin, 2000). However, given that images and image location were randomized, we would expect these potential effects to be minimal in the present study. Moreover, because emotional images were paired with neutral images and not other emotional images (i.e., positive–negative pairs), this approach diminishes concerns that our reported positive vs. negative gaze ratios were confounded by having to choose between viewing the negative or positive image.^2^ Lastly and relatedly, though naturalistic images achieve higher generalizability than more basic stimuli, the images may have differently evoked self-referential memories across participants. These self-referential effects could have subsequently influenced emotional processes, given that emotion-related effects are often amplified when self-relevance of a stimulus is high (Herbert et al., 2011). As such, obtaining self-relevance ratings may have revealed additional moderating effects on attentional biases as they relate to age, depression, and/or dispositional ER use.

In conclusion, our study provides support for attentional biases toward emotional information, but indicates that these biases shift depending on age and dispositional ER preferences. Depression may also play a meaningful moderating role that additional studies should corroborate. Specifically, a positivity bias (more time viewing positive images, less time viewing negative images) increased with age, but only among participants who preferred reappraisal over suppression and were not depressed. This finding extends prior work that suggests not a general shift in ER success across age, but rather a change in the relationship between ER implementation and attention toward emotional content. As humans age, they become more flexible in shifting from negative to more positive information during reappraisal (Bebko et al., 2011; Noh et al., 2011), particularly when the individual has the freedom to attend to positive information. Our results generally support the core principles of the SAVI model of affective aging, given that particular competency strengths (daily ERQ reappraisal use) and vulnerabilities (depression) moderate the age-associated attentional biases (Charles, 2010). Ultimately, these results emphasize the need to consider multiple individual difference factors that influence attention toward emotional content, as such biases are not universal.

## Author's note

The study design was preregistered as NCT03207503 at https://clinicaltrials.gov.

## Data Availability

The datasets presented in this study can be found in online repositories. The names of the repository/repositories and accession number(s) can be found below: https://osf.io/qkmt5/.

## Footnotes

^1^All participants also completed a third iteration of the BDI after the eye-tracking task that immediately preceded an MRI task not reported in this manuscript. For this participant, the third BDI assessment once again yielded a depressed status (BDI score = 18).

^2^We followed recommendations from Livingstone and Isaacowitz (2017) to include neutral images in affective eye-tracking paradigms in order to disentangle pro-positive versus anti-negative (or pro-negative versus anti-positive) effects.
