# Supplementary material for: Depression and emotion regulation strategy use moderate age-related attentional positivity bias
Source: Front Psychol. 2024 Dec 16;15:1427480. doi: 10.3389/fpsyg.2024.1427480 (PMC11682907; doi:10.3389/fpsyg.2024.1427480)
Supplement: Supplementary file 1 [file Data_Sheet_1.docx]

***Supplementary Material***

**Depression and Emotion Regulation Strategy Use Moderate Age-Related Attentional Positivity Bias**

Leonard Faul^☨^, Lucas Bellaiche^☨^, David J. Madden, Moria J. Smoski^^^, Kevin S. LaBar^^^*

^☨^These authors share first authorship

^These authors share senior authorship

***Correspondence:** klabar@duke.edu

**
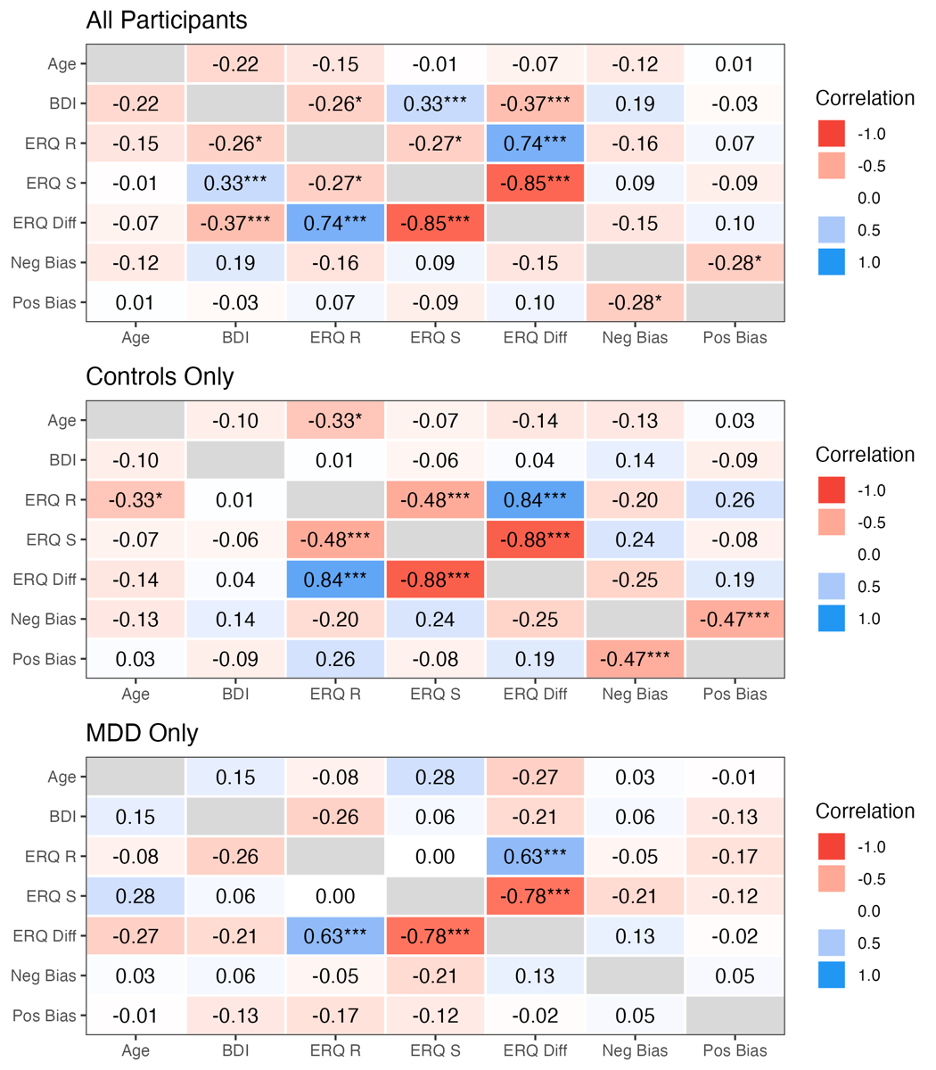
**

**Figure S1. Summary of Correlations Among the Primary Variables of Interest.** ERQ R (Reappraisal), ERQ S (Suppression), ERQ Diff (ERQ Preference Score), Neg Bias (Attentional Bias to Negative vs. Neutral Images), Pos Bias (Attentional Bias to Positive vs. Neutral Images). ******p* < .05, ***p* < .01, ****p* < .001 (uncorrected for multiple comparisons).


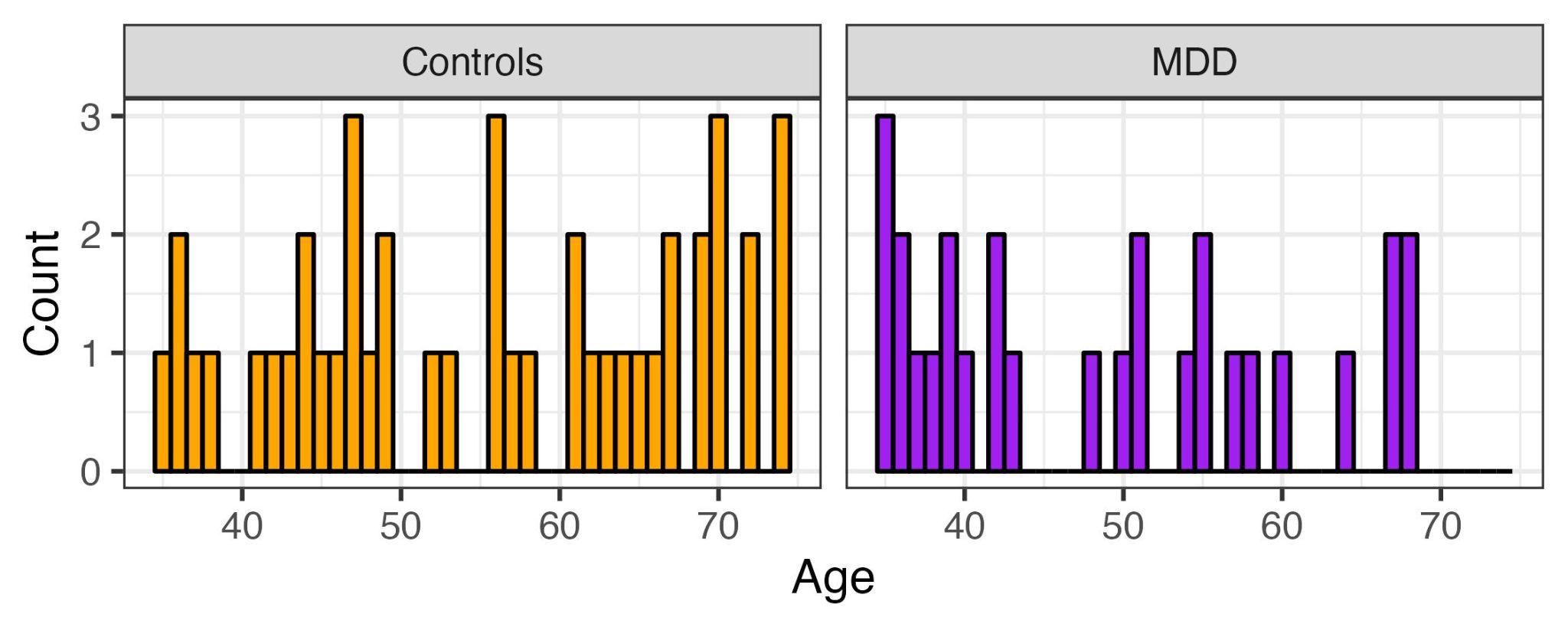


**Figure S2. Participant age distribution.** Histogram showing the ages of included participants, separated by MDD status.

*Sensitivity Analysis*

We performed a sensitivity analysis in G*Power for a within-between interaction design, again using the “*as in Cohen*” effect size specification. We specified four groups of participants (1: younger age + low reappraisal preference, 2: younger age + high reappraisal preference, 3: older age + low reappraisal preference, and 4: older age + high reappraisal preference), and two repeated measurements (two emotion conditions: positive and negative). This design approximates the three-way interaction we ultimately tested (age, condition, and ER preference), although it ignores our use of continuous measures for age and ER preference. To achieve power of 0.8 with such a design requires a large effect size of *f* = 0.52 for 44 participants (control group) and *f* = 0.68 for 28 participants (MDD group).

It is important to note that our use of linear mixed-effects regression with all available eye-tracking data and continuous between-subject measures (i.e., age and ERQ preference scores) allowed us to conduct more sensitive analyses to detect such effects compared to grouping the data as indicated above. Therefore, the calculated effect size from the sensitivity analysis is a considerable overestimation, as dichotomizing variables significantly reduces power (Altman & Royston, 2006). In conclusion, then, our design and analytical approach is appropriate to detect large effect sizes as specified by Cohen (1988), but may be underpowered to detect smaller effect sizes.

Altman, D. G., & Royston, P. (2006). The cost of dichotomising continuous variables. BMJ (Clinical research ed.), 332(7549), 1080. <https://doi.org/10.1136/bmj.332.7549.1080>

Cohen, J. (1988). Statistical Power Analysis for the Behavioral Sciences (2nd ed.). Routledge. <https://doi.org/10.4324/9780203771587>


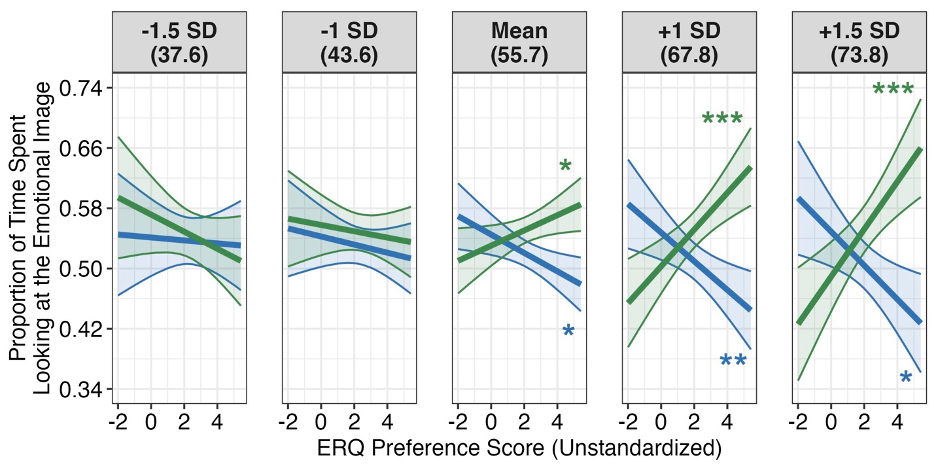


**Figure S3. Emotion Regulation Style Moderates Age-Related Positivity Biases in Non-Depressed Individuals (Alternate View of the Figure 3B Interaction Effect).** The relationship between ERQ preference score and dwell times at specific levels of age, only for healthy controls. Values in parentheses indicate the age value at each level from –1.5 to +1.5 SD (standardized within controls). Plots depict estimated marginal trends from the linear mixed effects model and 95% confidence intervals/bands. **p* < .05, ***p* < .01, ****p* < .001.

**
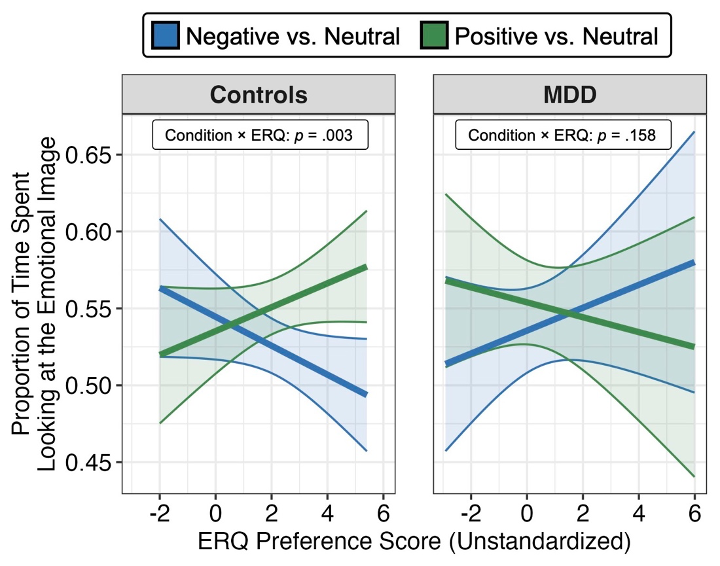
**

**Figure S4. Only Healthy Control Participants Exhibit a Significant Relationship Between ERQ Preference Scores and Dwell Times.** We observed a significant three-way interaction of BDI, condition, and ERQ preference (controlling for age effects; F_1,2091.44_ = 8.065, *p* = .005), which we unpacked by examining the two-way interaction of condition and ERQ preference when estimated marginal trends were averaged across the range of BDI scores for control (0-8) and MDD (10-47) participants. Note that similar effects were observed when using group assignment instead of BDI scores in the analyzed model. Plots depict estimated marginal trends obtained from the full linear mixed effects model (controlling for all age effects and interactions) and are shown with 95% confidence intervals.

*Separate Analyses with Reappraisal and Suppression Scores*

Exploratory assessments that separately examined reappraisal and suppression use (instead of a difference score) revealed similar findings as detailed in the main manuscript. Among control participants, the three-way interaction of condition, age, and ER remained significant for both reappraisal use (F_1,1275.35_ = 9.422, *p* = .002) and suppression use (F_1,1273.95_ = 4.334, *p* = .038). All effects in the MDD group remained non-significant (*p* > .05*).*
